# P-2079. Integrated Obstetric and Addiction Care Promotes Antepartum STI Screening and Poses Opportunities for Preventing the Vertical Transmission of HIV, HCV, and Syphilis

**DOI:** 10.1093/ofid/ofaf695.2243

**Published:** 2026-01-11

**Authors:** Lindsey E Hastings, Claudette Poole, Rachel Sinkey, Ellen Eaton

**Affiliations:** University of Alabama at Birmingham/Children's of Alabama, Birmingham, AL; University of Alabama at Birmingham, Birmingham, Alabama; University of Alabama at Birmingham, Birmingham, Alabama; University of Alabama, Birmingham, Birmingham, Alabama

## Abstract

**Background:**

Substance use in pregnancy can disrupt routine prenatal care and is linked with behaviors known to increase risk of sexually transmitted (STI) and bloodborne infections. This syndemic is challenging to address in pregnant patients, who face unique barriers to accessing substance use treatment. Dedicated obstetric programs may increase opportunities for STI screening and linkage to specialist care—allowing for timely treatment and prevention of vertical transmission.

**Table 1:**
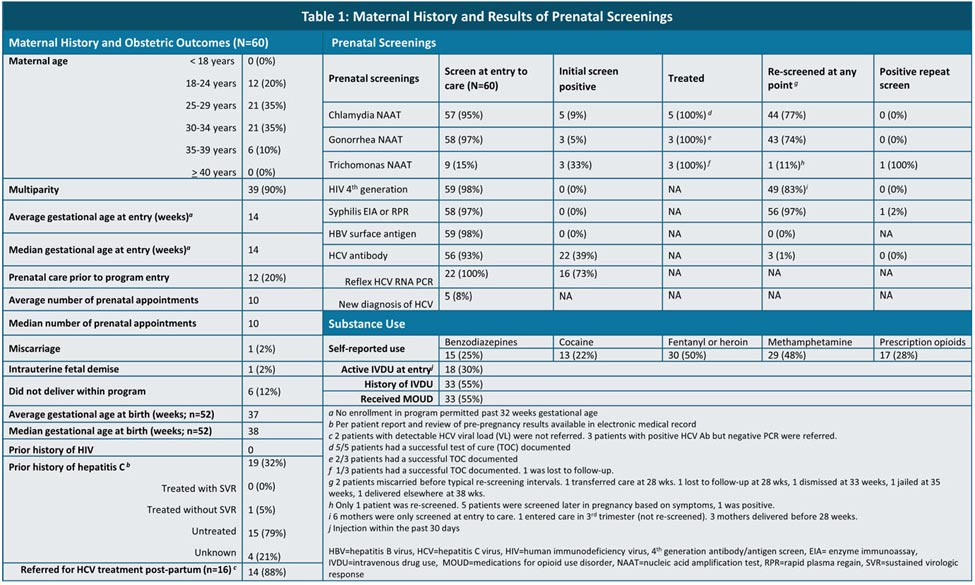
Maternal History and Results of Prenatal Screenings

**Table 2:**
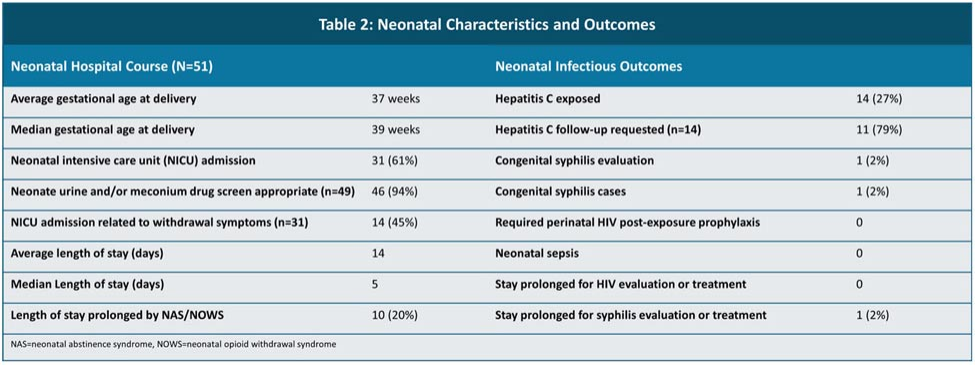
Neonatal Characteristics and Outcomes

**Methods:**

We identified 60 pregnant people followed by the Comprehensive Addiction in Pregnancy Program (CAPP) at the University of Alabama at Birmingham (UAB) between 2018-2022. All enrolled at < 32 weeks gestation and had active substance use. Electronic medical records (EMR) were manually reviewed for completion of guideline-recommended STI, HIV, HBV, and HCV screenings and STI treatment rates. 51 delivered at UAB and had infant charts available for review of birth and infectious outcomes.

**Results:**

Most individuals entered care by the 2^nd^ trimester and attended an average of 10 prenatal appointments. Entry screening rates were > 95% for gonorrhea, chlamydia, syphilis, and HIV (Table 1). Nearly 75% were re-screened for gonorrhea and chlamydia prior to delivery. 83% of patients were re-screened for HIV. 97% were re-screened for syphilis at least once, with 85% screened in the 3^rd^ trimester and 91% screened at delivery. One case of primary syphilis was diagnosed, resulting in a case of highly probable congenital syphilis.

19 (32%) reported a history of HCV, 93% were screened on entry, and 4 new diagnoses of HCV were made. The majority were referred for post-partum HCV treatment (Table 1). 27% of infants were deemed HCV-exposed, with 11 (79%) having HCV follow-up requested (Table 2).

**Conclusion:**

Pregnant persons enrolled in CAPP had high rates of guideline concordant STI screening and referral for HCV treatment. Compared with several national cohorts including pregnant Medicaid enrollees in the U.S. South, participants also had a higher rate of syphilis screening. Integrated obstetric and addiction care models have the potential to boost STI screenings and increase MOUD uptake, possibly serving as a harm reduction strategy for perinatal infections and offering another tool for combatting the congenital syphilis epidemic.

**Disclosures:**

Ellen Eaton, MD, MPH, Gilead: Honoraria

